# Comparative eco-physiology revealed extensive enzymatic curtailment, lipases production and strong conidial resilience of the bat pathogenic fungus *Pseudogymnoascus destructans*

**DOI:** 10.1038/s41598-020-73619-7

**Published:** 2020-10-05

**Authors:** Tereza Veselská, Karolína Homutová, Paula García Fraile, Alena Kubátová, Natália Martínková, Jiří Pikula, Miroslav Kolařík

**Affiliations:** 1 Laboratory of Fungal Genetics and Metabolism, Institute of Microbiology, Czech Academy of Sciences (CAS), Vídeňská 1083, 14220 Prague, Czech Republic; 2 Department of Botany, Faculty of Science, Charles University, Benátská 2, 12801 Prague, Czech Republic; 3 Institute of Vertebrate Biology, Czech Academy of Sciences (CAS), Květná 8, 60365 Brno, Czech Republic; 4 Department of Ecology and Diseases of Game, Fish and Bees, Faculty of Veterinary Hygiene and Ecology, University of Veterinary and Pharmaceutical Sciences Brno, Palackého třída 1946/1, 61242 Brno, Czech Republic

**Keywords:** Microbial ecology, Pathogens

## Abstract

The genus *Pseudogymnoascus* encompasses soil psychrophilic fungi living also in caves. Some are opportunistic pathogens; nevertheless, they do not cause outbreaks. *Pseudogymnoascus destructans* is the causative agent of the white-nose syndrome, which is decimating cave-hibernating bats. We used comparative eco-physiology to contrast the enzymatic potential and conidial resilience of *P. destructans* with that of phylogenetically diverse cave fungi, including *Pseudogymnoascus* spp., dermatophytes and outdoor saprotrophs. Enzymatic potential was assessed by Biolog MicroArray and by growth on labelled substrates and conidial viability was detected by flow cytometry. *Pseudogymnoascus* *destructans* was specific by extensive losses of metabolic variability and by ability of lipid degradation. We suppose that lipases are important enzymes allowing fungal hyphae to digest and invade the skin. *Pseudogymnoascus destructans* prefers nitrogenous substrates occurring in bat skin and lipids. Additionally, *P. destructans* alkalizes growth medium, which points to another possible virulence mechanism. Temperature above 30 °C substantially decreases conidial viability of cave fungi including *P. destructans.* Nevertheless, survival of *P. destructans* conidia prolongs by the temperature regime simulating beginning of the flight season*,* what suggests that conidia could persist on the body surface of bats and contribute to disease spreading during bats active season.

## Introduction

*Pseudogymnoascus destructans* (Pseudeurotiaceae, Ascomycota)^1^ is a fungus that infects the skin of hibernating bats and causes the disease called the white-nose syndrome (WNS)^2–4^. This fungus is native to Eurasian bat hibernacula, where it behaves as a not too virulent cutaneous pathogen. It causes sporadic mortality and health deterioration, but not apparent changes in bat populations^5,6^. This fact, together with the high genetic diversity of the European *P. destructans* populations, suggests its long co-evolution with its hosts^6–10^. A single *P. destructans* clone was introduced from Europe or Asia to the United States, where it encountered naïve hosts and has been causing massive mortality observed since 2006^11,12^. Despite of its clonal growth *P. destructans* manifests some fenotypical divergence^13,14^.

*Pseudogymnoascus* *destructans* infects naked parts of the bat skin, such as the muzzle, ears and flight membranes, where it forms cup-like epidermal erosions and ulcerations. Unlike dermatophytes^15^, *P. destructans* invades no only the epidermis, but grows into deeper parts of the skin, the dermis^2,15–17^. The ability to make a lesion is the same between *P. destructans* strains in the United States and Europe^18^. The lethal progress described as WNS is thus a result of complex interactions of variables, which are under debate^19–22^. Despite availability of extensive studies on *P. destructans* biology, the decided answer about its virulence and biology still missing and further research is desired to get closer. *P. destructans* shares some virulence factors with dermatophytes^23,24^, such as subtilisin-like proteases degrading collagen^25^, but lacks others, for example keratinase^26^. Comparative studies^16,24^ have identified siderophore secretion as virulence factor^19,27^, which is similar to fungi forming deeper mycotic infections^28^, where siderophores play an important role in the pathogenesis. *Pseudogymnoascus* *destructans*, in contrast to non-virulent *Pseudogymnoascus* species, is also unique in its overproduction of riboflavin, the role of which in virulence needs to be assessed further^19^. The WNS transcriptome revealed over fifty genes that are upregulated during the infection process on the side of the fungus^29–31^. They include numerous secreted proteases (including destructins and a homolog of the *Aspergillus fumigatus* major allergen Aspf2), heat shock proteins, ureases, metal-binding siderophores, enzymes responsible for fatty acid utilization and protein kinases.

WNS is spread by bat-to-bat transmission during hibernation^32^ and by infections from environmental pool of the cave sediments^33,34^. Non-pathogenic *Pseudogymnoascus* species are common soil fungi also living outside of hibernacula^1^. To the contrary, alternative habitats for *P. destructans* are so far unknown. It is unclear which physiological constraints restrict this fungus to the cold and humid habitats of caves. *P. destructans* is a psychrophilic fungus growing at temperatures between 3 and 19 °C, with an optimal range of 12–15 °C. Its growth stops at 10 °C and above 20 °C^35–37^. The optimal relative humidity for the growth of *P. destructans* is around 80%, which is the moisture level found in bats’ hibernacula and their body surface during hibernation^15^. Its psychrophilic nature enables bats to clear any visible infection during summer, when ambient temperatures exceed the upper limit for *P. destructans* growth. Nevertheless, we have only sparse information about its conidial resilience^13,38,39^. It therefore remains unknown whether *P. destructans* conidia can disseminate on the body surface of bats during the active flight season to new hibernacula and participate in the spreading of the disease.

The goal of our study was to compare phenotype of *P. destructans* with that of (1) other *Pseudogymnoascus* species to find traits specific to *P. destructans.* We propose that these traits are potentially under selective pressure and are possible virulence factors, (2) cave fungi to identify common traits related to growth in cave habitat and thus not directly linked with *P. destructans* pathogenesis and (3) dermatophytes which are pathogens and thus shared traits could be important in *P. destructans* virulence. Our study combines knowledge of prior studies on virulence factors of *P. destructans* and compares phenotype of *P. destructans* with phenotype of ecologically related species, which enables to estimate selective pressure leading to pathogenicity of *P. destructans*.

## Results

### Biolog analysis

**Figure 1:**
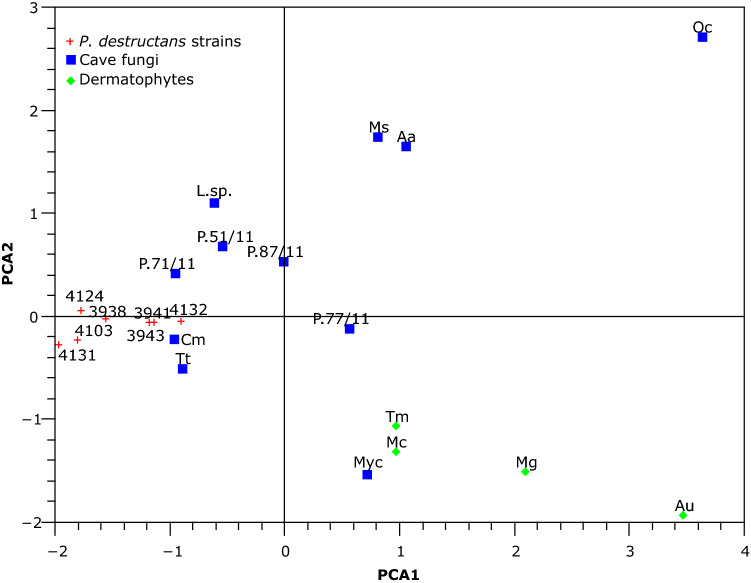
PCA analysis showing dissimilarities in nutrient metabolism between *P. destructans*, other cave fungi and dermatophytes on Biolog MicroPlates. The plotted PCA axes describe 52.3% of the variability in the data. *Tt* *Trichophyton terestre*, *Cm* *Chrysosporium merdarium*, *L.sp*. *Leotiomycetes* sp. CCF6130, *P.71/11* *Pseudogymnoascus* sp. 2 AK71/11, *P.77/11* *P*. sp. 2 AK77/11, *P.87/11* *P*. sp. 4 AK87/11, *P.51/11* *P*.sp. 1 AK51/11, *Ms* *Metapochonia suchlasporia*, *Aa* *Aspergillus askiburgiensis*, *Oc* *Oidiodendron cerealis*, *Myc Myotisia cremea* CCF5407, *Tm* *T. mentagrophytes*, *Au* *Arthroderma uncinatum,* *Mc* *Microsporum canis*, *Mg* *M. gypseum.*

Analyzed ecological groups formed well-defined clusters on PCA analysis (Fig. 1), only a cave inhabiting fungus *Myotisia cremea* (Onygenales), which lives on the bats guano, clustered with dermatophytes. Based on one-way NPMANOVA analysis, *P. destructans* significantly differed in growth potential from dermatophytes on each of the Biolog MicroPlates tested (*p* < 0.05). *Pseudogymnoascus destructans* was also distinct from cave fungi (*p* < 0.05) except growth on sulfur and phosphorus sources (*p* = 0.75). The main differences between these groups resided in the limitation of *P. destructans* to only a few nitrogen substrates and its increased growth potential on nutrient supplements (Supplementary Dataset online). *Pseudogymnoascus destructans* was significantly distinct from *Pseudogymnoascus* species on carbon and nutrient supplements (*p* < 0.05). *Pseudogymnoascus* species were similar to cave fungi on all microplates (*p* > 0.7). When all microplates were integrated into one analysis (Fig. 1), *P. destructans* was significantly separated from cave fungi, *Pseudogymnoascus* spp. and dermatophytes (*p* < 0.02), and dermatophytes differed from cave fungi (*p* = 0.01). *Pseudogymnoascus* spp. were significantly different from *P. destructans* (*p* < 0.02) and were similar to cave fungi (*p* = 1).

**Figure 2:**
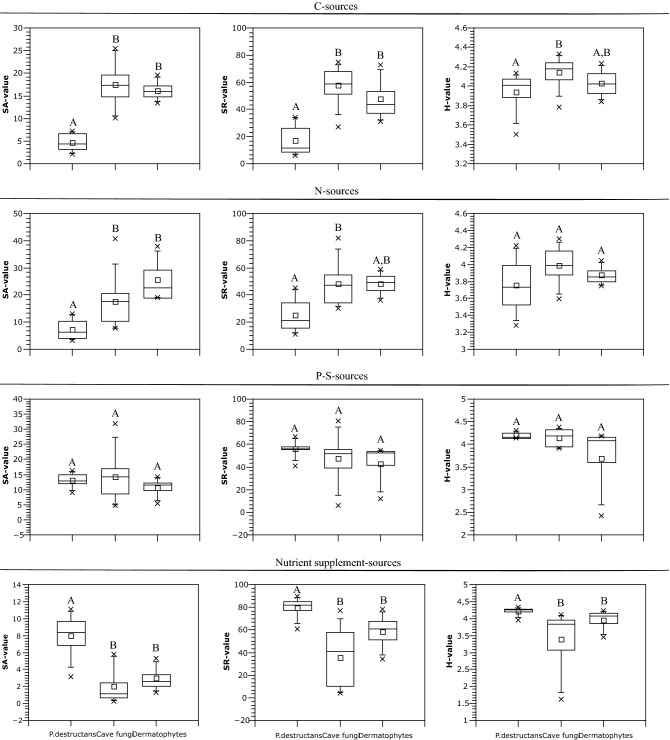
Growth characteristics on Biolog MicroPlates described by SA, SR and H values. Different letters indicate significant differences between groups and same letters indicate similarity based on Kruskall-Wallis statistics supplemented with Mann–Whitney pairwise comparison and Bonferroni correction.

*Pseudogymnoascus destructans* was remarkable for its relatively low SA and SR but high H values on microplates containing carbon and nitrogen sources (Fig. 2). This means that even if *P. destructans* growth is supported by only a few carbon and nitrogen sources, it also preserves a weak growth capability on most of them. The most growth-supporting carbon and nitrogen substrates for *P. destructans* were the lipid Tween 80, glycogen, and urea, allantoin, uric acid and ammonia (Table 1, Supplementary Table S1 online). Cave fungi and dermatophytes also favoured these nitrogen substrates. *P. destructans* grew well on nutrient supplements, especially on amino acids. All groups had similar growth potential on phosphorus and sulphur substrates. Cave fungi displayed versatility in carbon utilization (highest value of SA, SR and H). Dermatophytes were specific in their preference for nitrogenous carbon sources (mostly amino acids and N-acetyl-D-glucosamine).

**Table 1:** Top ten utilized substrates by *P. destructans* on each Biolog MicroPlates, nutrient sources in parentheses.

FF (C)	PM3 (N)	PM4 (S + P)	PM5 (nutrient supplements)
Tween 80	Gly-Gln	Cytidine-2-monophosphate	L-lysine
D-mannose	Urea	O-phosphoryl-ethanolamine	L-leucine
Gentibiose	Guanosine	O-phospho-L-serine	L-valine
D-trehalose	Gly-Asn	6-phospho-gluonic acid	Orotic acid
D-fructose	Gly-Glu	Guanosine-2-monophosphate	L-isoleucine + L-valine
α-D-glucose	Allantoin	2-deoxy-D-glucose 6-phosphate	D,L-α,δ-diamino-pimelic acid
Glycogen	Ala-Gly	D-3-phospho-glyceric acid	D-alanine
L-glutamic acid	L-glutamine	Carbamyl phosphate	L-tyrosine
Turanose	Ammonia	Uridine-5-monophosphate	L-cysteine
D-psicose	Uric acid	Guanosine-5-monophosphate	Trans-4-hydroxy L-proline

### Semi-quantitative extracellular enzymes activities

We compared the production of extracellular enzymes cleaving connective tissue between *P. destructans* and other *Pseudogymnoascus* species. We detected the production of lipases, elastases and collagenases. *Pseudogymnoascus destructans* was specific in the production of lipases and lack of elastases. There was no difference in the production of collagenases (Table 2).

**Table 2:** Semi-quantitative extracellular enzymes activities indicate the presence of lipases and lack of elastases in *Pseudogymnoascus destructans* compared to *P*. spp.

Species	Strain	Lipases (olive oil)	Elastases pH 5.5	Elastases pH 7	Elastases pH 8.5	Colagenases pH 5.5	Colagenases pH7	Colagenases pH 8
*P. destructans*	20,631-21^ T^	** + **	−	−	−	+	+	+
	CCF3941	** + **	−	−	−	+	+	+
	CCF3943	** + **	−	−	−	+	+	+
	CCF4103	** + **	−	−	−	+	+	+
	CCF4987	** + **	−	−	−	+	+	+
	CCF4986	** + **	−	−	−	+	+	+
*P.* sp. 1	CCF5025	−	−	−	−	+	+	+
*P.* sp. 2	CCF5030	−	−	+ +	+	+	+	+
*P.* sp. 2	CCF5027	−	−	+ +	−	+	+	+
*P.* sp. 2	CCF5029	−	−	+ + +	+ + +	+	+	+
*P.* sp. 3	CCF5026	−	−	+ +	+ +	+	+	+

− Activity absent, + / +  + / +  +  + detected activity from weak to strong.

### Medium alkalization by urea degradation

**Figure 3:**
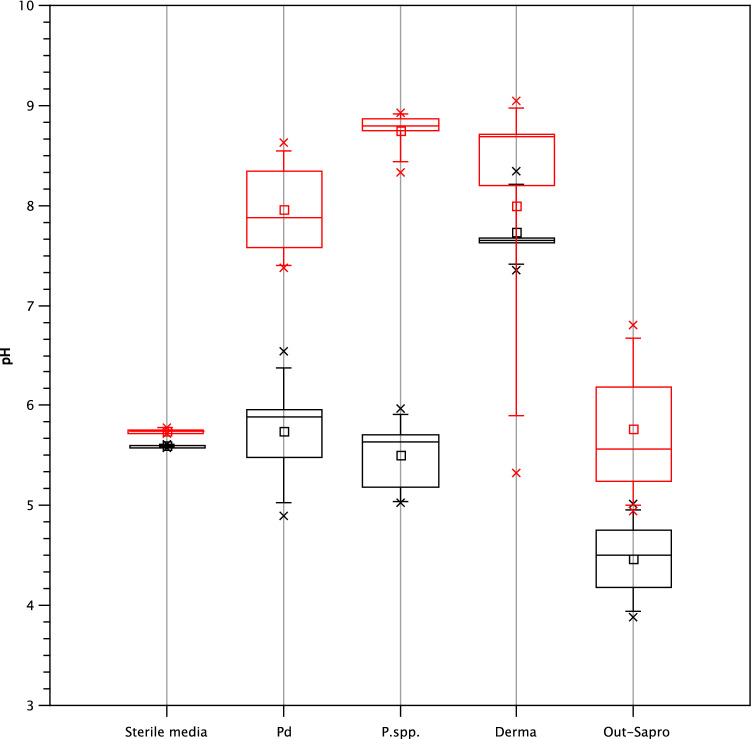
Increase of growth medium pH caused by urea metabolism. The Sabouraud medium is plotted in black and the Sabouraud medium supplemented with urea is plotted in red. *Pd* *Pseudogymnoascus destructans*, *P.spp.* *Pseudogymnoascus* species, *Derma* dermatophytes, *Out-Sapro* outdoor saprotrophs.

*Pseudogymnoascus* species including *P. destructans* significantly increase pH in Sabouraud medium supplemented with urea (permutation test, *p* = 0.0001) (Fig. 3, Supplementary Table S2 online). Dermatophytes increased the pH of both media equally (*p* = 0.8), except for *Arthroderma uncinatum* which only alkalized medium without the presence of urea. Outdoor saprotrophs alkalized none of media analysed (*p* = 0.29). We detected a slight pH decrease (< 0.2) in both of sterile media over the course of the experiment. This means that the detected changes in the pH of inoculated media were the result of fungal growth.

### Conidial viability under stressful conditions

**Figure 4:**
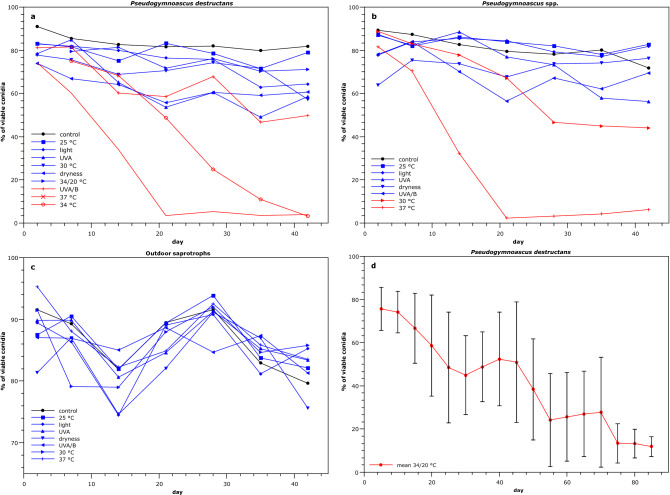
Test of conidial viability under stressful conditions. A-C: Conidial viability affected by variable stress factors for 42 days. D: prolonged effect of periodic temperature switches from 34 to 20 °C (12 h period, 85 days) on conidial viability of *P. destructans*. Mean values with standard deviations for seven strains are presented. Stress factors with a minor effect are plotted in blue. Stress factors causing a decrease in conidial viability under 50% are marked red.

We evaluated conidial viability under stressful conditions over a period of 42 days for nine *P. destructans* strains, three non-pathogenic *Pseudogymnoascus* species and two air-borne *Aspergillus* species (Fig. 4, Supplementary Table S3 online).

All *Pseudogymnoascus* species, including *P. destructans*, were sensitive to temperatures higher than 30 °C. At the end of the incubation at 30 and 37 °C, conidial viability of *Pseudogymnoascus* spp. dropped to 44.1% and 6.3%, respectively. *P. destructans* conidia exhibited greater resilience to incubation in 30 °C, final viability of its conidia was 57.2%. Nevertheless, its conidial viability was less than 4% at temperatures between 34 and 37 °C. *P. destructans* was also mildly sensitive to UVA/B radiation, with 49.8% of viable conidia. Our next experiment traced the conidial viability of *P. destructans* strains over a 12-h period of repeated temperature switches from 20 to 34 °C. The changing of temperature markedly enhanced their conidial viability to 64.5%. Even after 85 days of exposure, their conidial viability remained between 6 and 17%, with a mean value of 11.1%. The *Aspergillus* species were resistant to all of the stressful conditions. Sensitivity to the dry conditions was similar across the tested species.

## Discussion

Our aims were (1) to ascertain the enzymatic potential and ability of environmental alkalization of *P. destructans*, to compare it with a phylogenetically diverse set of cave fungi (including non-pathogenic *Pseudogymnoascus* spp.), dermatophytes and outdoor saprotrophs, and (2) to characterize the viability of *P. destructans* conidia under stressful conditions. This enabled us to trace potential virulence factors of *P. destructans* and to propose the physiological constraints of *P. destructans* that limit its distribution to bat-inhabited caves. Information about conidial stress tolerance is necessary for a complex evaluation of the dissemination potential of *P. destructans* and the spreading of the white-nose syndrome. We found that *P. destructans* has extensively reduced metabolic potential compared to species tested, including other *Pseudogymnoascus* species. *P. destructans* produces lipases, which may be important for its pathogenicity. Conidia of *P. destructans* are more resilient than mycelium to harsh environmental conditions. Thus, it could mean that conidia contribute to disease spreading after bat departures from caves after winter.

The enzymatic profile of *P. destructans* was more similar to saprotrophic cave fungi than to dermatophytes. The similarity of *P. destructans* with cave fungi found in this study was expectable and points to its descent from the saprotrophic *Pseudogymnoascus* species. *P. destructans* is generally isolated directly from bats, but rarely also from sediments of abandoned caves^33^ where it grows on cellulose and protein/lipid substrates^40,41^. Our Biolog analyses indicate that *P. destructans* is able to use a broad range of carbon, nitrogen, phosphorus and sulfur sources, but its growth on most such sources is weak. This suggests that *P. destructans* could survive with low fitness on a variety of substrates. Nevertheless, faster-growing cave fungi probably outcompete *P. destructans* on these non-preferred substrates.

Despite the supposed saprophytic origin of *P. destructans*, our results from enzymatic and total Biolog assimilation profiles clearly separate *P. destructans* from cave fungi, including *Pseudogymnoascus* spp. Compared to cave fungi, *P. destructans* strains display intensive enzymatic curtailment on Biolog MicroPlates. We propose that it could be result of *P. destructans* long-term specialization to a specific niche with limited set of available nutrients, which led to subsequent selective loses of metabolic pathways. This supports previous evidence that the metabolism of *P. destructans* is restricted compared to other *Pseudogymnoascus* species^38,42–44^ and confirms the long co-evolution between this pathogen and its hosts.

Both dermatophytes and *P. destructans* grow on mammal skin, and thus have similar nutrient sources available for growth. Our sampling unable us to draw decided conclusions; however, it seems that they have different nutritive strategy. While dermatophytes prefer nitrogenous substrates, *P. destructans* is rather specialized on lipid substrates. *P. destructans* even differs from analyzed cave fungi, including non-pathogenic *Pseudogymnoascus* spp., that prefer saccharides as carbon sources. These results correspond with prior findings of lipid degradation by *P. destructans*^40,45^. We propose that *P. destructans* preference of lipids is one of virulence factors, which enables *P. destructans* to colonize bat tissues. Lipases enable fungi to damage epidermal and epithelial tissue. They also influence fungal growth, adherence and dissemination inside the host^46^. Our comparative eco-physiological approach proposes outstanding role of lipid and fatty acid degradation in the virulence of *P. destructans,* as already noted by Donaldson et al.^29^ based on transcriptomic data. Given the facts that *P. destructans* lacks keratinolytic activity and that phospholipids are a major component of all cell membranes, lipases are probably the most important enzymes allowing fungal hyphae to digest and invade the integumentary structures of infected skin, forming cup-like erosions diagnostic of the white-nose syndrome.

Similarly to cave fungi including *Pseudogymnoascus* spp., *P. destructans* prefers urea, allantoin, ammonia and uric acid as nitrogen sources. These substances are products of the metabolic breakdown of nitrogenous compounds. Beside occurrence in sediments, these substances are also contained in urine and sweat. In bat skin, they are typical components of sweat glands, where hyphal growth of *P. destructans* was described^2^ and thus where are easily accessible for its nutrition. Beside nitrogen metabolism, urease has a versatile functions in plants, animals and microorganisms, as is reviewed in^47^. It participates in fungal pathogenesis^48,49^, for example by production of ammonia which cause environmental alkalization^50^ and subsequent spontaneous creation of reactive oxygen species^51^ and tissue damage. We detected an increase in pH caused by the degradation of urea during cultivation of *P. destructans*. Nevertheless, we were not successful in direct pH measurements in bat necrosis, thus further experiments are needed to examine the role of pH manipulation in virulence of *P. destructans*.

Similarly to other *Pseudogymnoascus* species, *P. destructans* produces collagenases that erode connective tissues and enable fungi to penetrate the skin of bats. Collagenase production is well known in *P. destructans*^25,52^, but from our data we propose that it is probably a preadaptation present in the whole genus, not an adaptation primarily responsible for the behavior of *P. destructans.* This corresponds with the fact that *P. destructans* has an equal level of protease expression (including that of destructins) in agar culture and on the wings of bats^29^. Similarly, subtilisin-like serine proteases, whose role in virulence is not yet clear, are present both in *P. destructans* and *P. pannorum*^25^. Our results presume that *P. destructans* lacks the ability to cleave other constitutive proteins in the skin, elastin and keratin, which may suggests that the pathogen does not use enzymatic skin degradation primarily for nutrient acquisition. Thus, we propose that other nutrient sources like lipids, urea and various nitrogenous sources found in sweat glands are targets for assimilation by *P. destructans*.

Preceding studies have shown that temperatures above 20 °C^37^ and relative humidity lower than 70%^53^ impedes the mycelial growth of *P. destructans*. Our viability tests revealed that conidia of *P. destructans* are more resilient to higher temperatures and lower humidity than its mycelium. Recent study^39^ has proposed that conidia of *P. destructans* could germinate even after exposition to elevated temperatures. Our simulation of temperature changes on the surface of a bat’s body after its departure from a cave indicates even more prolonged conidial viability of *P. destructans* at higher temperatures. In vitro conditions are far away from that in nature, where more factors are combined and could have cumulative negative effect on viability of conidia. Thus, we cannot foresee how long conidia of *P. destructans* stay viable on the bat body surface after its departure from cave. However, can state that conidia of *P. destructans* are much more resilient to harsh environmental conditions than its mycelium. As conidia are the primary dissemination agent of fungi, we wonder if they might be able to survive on the surface of the bat body reaching the next hibernation site.

## Conclusion

Analyzed cave fungi, despite being phylogenetically unrelated, represent a metabolically well-defined group. Among them, *Pseudogymnoascus destructans* evinces specific physiological profile. We suggest that it is a result of its strong specialization to life on bat skin and pathogenicity. *Pseudogymnoascus destructans* is specific by loss of redundant metabolic variability and production of lipases. We propose that lipases are probably the most important enzymes allowing fungal hyphae to digest and invade the integumentary structures of infected skin. The future investigations of virulence could test our hypothesis by specific knock-out of these genes. *Pseudogymnoascus destructans* conidia show great viability potential under various stressful condition, which offers possibility of their inactive persistence on the body surface of bats during their active season. These resting conidia are maybe able to germinate when bats reach their hibernation sites, where environmental conditions enable the growth of *P. destructans.* This could mean that once infected bats might serve as a reservoir and vector for the spread of the disease to new hibernacula.

## Methods

### Fungal material and cultivation

The type strain from the USA and nine European strains of *P. destructans* strains covering different haplotypes and mating types identified according to Zukal et al.^6^ were used. Representatives of typical non-pathogenic cave-inhabiting fungi (8 genera from four Ascomycota orders, 16 strains) and dermatophytes (2 genera, 5 strains) or saprotrophic fungi (2 genera, 3 strains) living outside of caves were chosen for comparison (Tables 3, 4). Relatively low representation of dermatophyte and outside living saprotrophic strains prevents general conclusion about their physiology. Nevertheless, it was not attempt of our study. Inclusion of these species served us for suggestions of potential selective pressures that could play a role in formation of *P. destructans* physiology*.* Fungal isolates were identified by ITS rDNA barcoding using the methods of Kolařík et al.^54^, and their sequences were deposited in the EMBL sequence database. Cultures were deposited in the Culture Collection of Fungi (abbreviations CCF or AK) at the Department of Botany, Faculty of Science, Charles University in Prague. *Pseudogymnoascus* species including *P. destructans* were grown in Petri dish containing glucose yeast extract agar (GYEA, 20 g glucose, 5 g yeast extract, 15 g agar, 1 l distilled water) at 10 °C unless stated otherwise. Cave fungi *Trichophyton terrestre* AK44/09, Leotiomycetes sp. CCF6130 (88/11) and *Chrysosporium merdarium* CCF6131 (AK91/11) were grown on 4° malt agar (MA) at 10 °C. Other fungi were cultivated on 4° MA at 25 °C.

**Table 3:** Fungal strains used in this study.

Group	Fungus	Source	Sequence accession number	Reference
*Pseudogymnoascus destructans* (Leotiomycetes)	CCF3938	CZE, Solenice, *Myotis myotis*, 2010	HM584954	^66^
	CCF3941	CZE, Bohemian Karst, Malá Amerika mine, *Myotis myotis*, 2010	HM584956	^66^
	CCF3943	CZE, Stříbro, *Myotis myotis*, 2010	HM584957	^66^
	CCF4103	CZE, Herlíkovice, Krkonoše Mts., *Pleurotus auritus*, 2011	LN852366	^19^
	CCF4124	CZE, Horní Albeřice, Krkonoše, *Myotis myotis*, 2011	KJ938421	^9^
	CCF4131	CZE, Vyškov u Chodové Plané, *Myotis myotis*, 2011	KJ938420	^9^
	CCF4132	CZE, Pernink, *Myotis myotis*, 2011	nd	^9^
	CCF4987	CZE, Kašperské Hory, *Myotis myotis*, 2014	LN871252	^6^
	CCF4986	Rusia, Ural mts., cave Smolinskaya, *Myotis dasycneme*, 2014	LN852359	^6^
	20631-21^T^	USA, Williams Hotel, NY, *Myotis lucifugus,* 2008	EU884921	^35^
Saprotrophic cave fungi	*Pseudogymnoascus* sp. 1 CCF5025^1^	CZE, Bohemian Karst, Alkazar tunnel, bat excrement, 2009	LN852360	^19^
	*P.* sp. 2 CCF5030^2^	CZE, Moravia, *Myotis myotis*, 2012	LN852361	^19^
	*P.* sp. 2 CCF5027^2^	CZE, cave sediment, Moravia, Javoříčske Caves, 2012	LN852363	^19^
	*P.* sp. 2 CCF5029^2^	CZE, Moravia, Javoříčske caves, cave sediment, 2012	LN852364	^19^
	*P.* sp. 3 CCF5026^3^	CZE, Moravian Karst, Sloupsko-Šošůvké Cave, *Rhinolophus hipposideros*, 2013	LN852365	^19^
	*P.* sp. 1 AK51/11^4^	CZE, Herlíkovice, Krkonoše, *Eptesicus nilssonii*	LN714595	^67^
	*P.* sp. 2 AK71/11^5^	CZE, Velká Amerika mine, sediment, 2011	Submitted to EMBL	This study
	*P.* sp. 4 AK87/11^6^	CZE, Bohemian Karst, Koněpruské jeskyně caves, sediment, 2011	Submitted to EMBL	This study
	*P.* sp. 2 AK 77/11^7^	CZE, Bohemian Karst, Velká Amerika mine, sediment, 2011	Submitted to EMBL	This study
	*Trichophyton terrestre* AK44/09 (Onygenales)	CZE, Bohemian Karst, Alkazar tunnel, excrement, 2009	LN714614	^67^
	*Myotisia cremea* CCF5407 (Onygenales)	CZE, Bohemian Karst, Malá Amerika mine, bat excrement, 2009	LT627243	^68^
	*Metapochonia suchlasporia* CCF6128 (Clavicipitaceae)	CZE, Bohemian Karst, Velká Amerika mine, sediment, 2011	Submitted to EMBL	This study
	*Leotiomycetes* sp. CCF6130 (= AK88/11)^8^	CZE, Bohemian Karst, Velká Amerika mine, sediment, 2011	Submitted to EMBL	This study
	*Chrysosporium merdarium* CCF6131 (AK 91/11) (Leotiomycetes)	CZE, Karlštejn castle, sediment in the castle well, 2011	Submitted to EMBL	This study
	*Oidiodendron cerealis* CCF3491 (Leotiomycetes)	CZE, Bedřichov, tunnel wall, 2004	ng	This study
	*Aspergillus askiburgiensis* CCF4085 (Eurotiales)	CZE, Bohemian Karst, Malá Amerika mine, WNS positive *Myotis myotis*, 2010	LN873940	^69^
Saprotroph from the outside of underground spaces	*Aspergillus luchuensis* CCF3984 (Eurotiales)	CZE, Praha, tea bag (Yerba maté ), 2010	FR727131	V. Hubka, unpublished
	*A. flavus* CCF3154 (Eurotiales)	Brno ČR, black papper, 1999	ng	
	*Penicillium oxalicum* 2315^T^ (Eurotiales)	USA, soil, Connecticut, 1914	HE651152	^70^
Dermatophyte	*Microsporum canis* CCF3443 (Onygenales)	CZE, Ostrava, human skin, 2003	ng	
	*Microsporum gypseum* CCF3100 (Onygenales)	CZE, Šumperk, human skin, 1998	ng	
	*Arthroderma uncinatum* CCF2907 (Onygenales)	CZE, Horní Počaply ČR, soil with industrial ash deposits, 1994	ng	
	*Trichophyton mentagrophytes* CCF3954 (Onygenales)	CZE, Pardubice, human skin, 2009	ng	
	*Trichophyton interdigitale* CCF4473 (Onygenales)	CZE, tinea corporis, human skin, 2012	LN736306	^71^

*CZE* Czech Republic.

^1^ITS rDNA identical with JX270356. “Clade L” sensu^1^.

^2^ITS rDNA identical with JX845296. “Clade B” sensu^1^.

^3^ITS rDNA identical with JX270432. “Clade J” sensu^1^.

^4^ITS rDNA 99% (466/467 bp) similarity with JX270356. “Clade L” sensu^1^.

^5^ITS rDNA 99% (570/571 bp) similarity with JX270614. “Clade B” sensu^1^.

^6^ITS rDNA 99% (884/894 bp) similarity with JX270621. Phylogenetic position outside of the clades delimited by^1^.

^7^ITS rDNA 99% (893/896 bp) similarity with JX270443. “Clade B” sensu^1^.

^8^Best BlastN hits (90% for ITS rDNA) are various *Botrytis* species (e.g. *Botrytis cinerea* strain CBS 261.71, MH860108).

**Table 4:** List of analysed species and used methods.

Group	Fungal strain	Biolog	Extracellular enzymes	pH test	Conidial viability test
*Pseudogymnoascu*s *destructans*	20631-21^T^	−	+	+	−
	CCF3938	+	−	−	+
	CCF3941	+	+	+	+
	CCF3943	+	+	+	+
	CCF4103	+	+	+	+
	CCF4124	+	−	−	+
	CCF4131	+	−	−	+
	CCF4132	+	−	−	+
	CCF4986	−	+	+	−
	CCF4987	−	+	+	−
Cave fungi	*Aspergillus askiburgiensis* CCF4085	+	−	−	−
	*Chrysosporium merdarium* CCF6131	+	−	−	−
	*Leotiomycetes* sp. CCF6130	+	−	−	−
	*Metapochonia suchlasporia* CCF6128	+	−	−	−
	*Myotisia cremea* CCF5407	+	−	−	−
	*Oidiodendron cerealis* CCF3491	+	−	−	−
	*Pseudogymnoascus* sp. 1 AK51/11	+	−	+	+
	*P.* sp. 1 CCF5025	−	+	+	−
	*P.* sp. 2 CCF5027	−	+	+	−
	*P.* sp. 2 CCF5029	−	+	+	−
	*P.* sp. 2 CCF5030	−	+	+	−
	*P.* sp. 2 AK71/11	+	−	−	+
	*P.* sp. 2 AK 77/11	+	−	−	+
	*P.* sp. 3 CCF5026	−	+	+	−
	*P.* sp. 4 AK87/11	+	−	−	−
	*Trichophyton terrestre* AK44/09	+	−	−	−
Saprotroph	*Aspergillus flavus* CCF3154	−	−	+	+
	*A. luchuensis* CCF3984	−	−	+	+
	*Penicillium oxalicum* 2315^ T^	−	−	+	−
Dermatophyte	*Arthroderma uncinatum* CCF2907	+	−	+	−
	*Microsporum canis* CCF3443	+	−	+	−
	*M. gypseum* CCF3100	+	−	+	−
	*T. interdigitale* CCF4473	−	−	+	−
	*T. mentagrophytes* CCF3954	+	−	+	−

+/− Analyses done/not done.

### Biolog analysis

Biolog MicroPlate for Filamentous Fungi (FF) and Biolog Phenotype Micro-Arrays (PM) (Biolog, Inc., Hayward, CA) were used to evaluate the assimilation profiles of carbon (FF), nitrogen (PM3B), phosphorus, sulphur (PM4A) and nutrient supplements (PM5) according to the manufacturer’s instructions. Fungal conidia from grown cultures were transferred into the inoculating fluid (0.25% Phytagel, 0.03% Tween 40) by rolling a swab across sporulating areas to get the final transmittance of 75 ± 2% or 62 ± 2% for FF and PM, respectively. Inoculated plates were incubated at convenient temperatures as is described in “Fungal material and cultivation” section, and absorbance at 750 nm was used to measure mycelial growth. Two technical replicates per strain were prepared for the FF plates and one replicate was prepared for the PM plates. The isolates analysed differed in their growth rate. For this reason, the last readings before reaching a growth plateau of substrate utilization were used for analysis.

The absorbance of the negative control was subtracted from all substrates within one plate and negative values were assigned a value of zero^55^. Some substrates were omitted from the analysis due to their low solubility (FF—B1, B3, G2, H10; PM3B—C1, G1; PM4A—A3, B9). Functional diversity was evaluated based on substrate activity (SA), substrate richness (SR)^56^ and the Shannon index (H)^57^. In brief, SA was defined as the sum of the optical densities of all substrates on one plate greater than zero. SR is defined as the number of substrates on a microtitre plate that exhibit an optical density greater than the threshold. The optical density of 0.1 was fixed as the threshold for FF and PM3B plates, and the optical density of 0.01 was fixed for PM4A and PM5 plates^58^.

### Extracellular enzyme detection

Lipolytic activities were detected using the rhodamine B assay described by^59^. Rhodamine B plates were made using the Czapek substrate as the basal medium with the addition of rhodamine B (0.0001% w/v) and olive oil (1%) as a lipid substrate. Inoculated plates were incubated at 4 °C for three weeks, as this temperature is similar to that in bat hibernacula. Fungal colonies with lipolytic activity showed an orange fluorescent halo under UV light.

For collagenase assays, strains were grown as described by^60^ for two weeks at 4 °C. An adaptation of the double-layer plate assay was employed for the detection of enzymatic activity^61^. Briefly, Petri dishes were filled with a bottom layer containing 15 ml of 0.7% (w/v) agarose in 50 mM citric acid/sodium citrate buffer (pH 5.5) and 50 mM phosphate buffer (pH 7 and pH 8), and then overlaid with 5 ml of the same media with the addition of 2% w/v collagen. The plates were then inoculated with 10 μl drops from the cultures of the strains in the Czapek medium supplemented with collagen. Undigested collagen was detected by Coomassie brilliant blue G staining^60^. An identical procedure, in which the collagen was replaced by elastin, was followed to test the production of elastases. Elastin hydrolysis was detected based on the observation of halos^62^.

### Medium alkalization by urea degradation

The ability to alkalize the growth medium by the degradation of urea was investigated by comparison of pH changes during cultivation on a liquid Sabouraud medium (40 g glucose, 10 g pepton, 1 l distilled water) versus a liquid Sabouraud medium supplemented with 50 mM of filter sterilised urea. Erlenmeyer flasks containing 150 ml of the medium were inoculated by fungal strains and pH was measured by a digital pH meter until the maximum value was reached. Non-inoculated sterile media were used as a control. *Penicillium oxalicum* CCF2315 served as a negative control because of its known acidification ability. *Pseudogymnoascus* species were cultivated at 15 °C for up to 5 weeks, dermatophytes and outdoor saprotrophs were cultivated at 25 °C for up to 3 weeks.

### Conidial survival under stressful conditions

We compared the conidial viability of *P. destructans* under stressful conditions with that of several non-pathogenic *Pseudogymnoascus* species living in caves and two air-borne *Aspergillus* saprotrophic species. The fungi were grown under their optimal growth conditions to attain sufficient conidial production. Petri dishes (one replication for each set of conditions) with well-established fungal cultures were exposed for up to six weeks to ten sets of physical conditions: in the dark at 25 °C, 30 °C, 34 °C and 37 °C; at 25 °C with white light, UVA, UVA plus UVB radiation, and in dryness (dark). UVA and UVB radiation, and white light was administered by a fluorescent tube lamp UV 11 W/G23-DZ (11 W, 350–410 nm), a fluorescent tube lamp Repti Glo 10.0 (20 W, T8, 10% UVB and 33% UVA) and a white light bulb (Philips, LUX BL, 11 W, 230–240 V, ~ 50/60 Hz, 2700 K), respectively. The influence of dryness was tested on conidia obtained by rolling a swab across a well-established culture. Swabs with conidia were then placed into glass jars containing silica gel and sealed with Parafilm to keep humidity below 20%. Conidial viability was evaluated weekly. The next experiment traced conidial viability of *P. destructans* strains during a 12 h period of repeated temperature switches from 20 to 34 °C for 85 days in the dark, by which we simulated temperature changes during the day and night on the body surface of bats after their departure from a cave, i.e. temperatures of homeothermy and daily torpor during the bats’ active season^63^.

### Evaluation of conidial viability by flow cytometry

**Figure 5:**
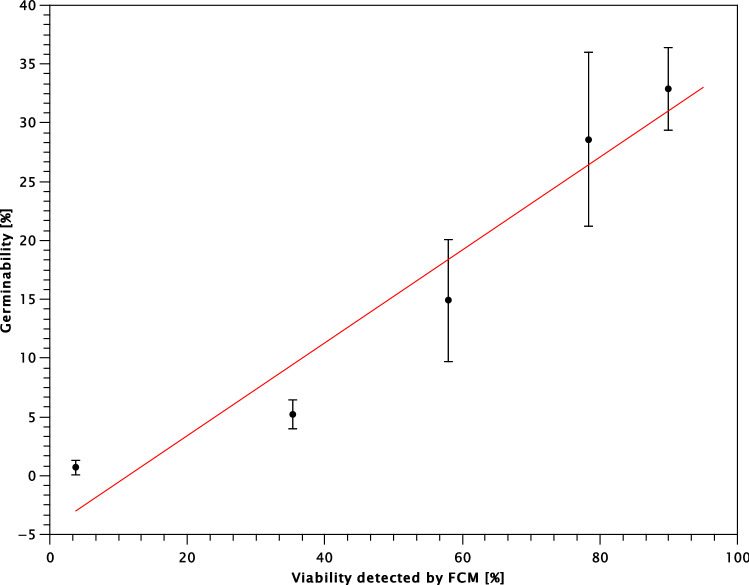
Strong correlation between proportion of germinated conidia on GYEA and proportion of viable conidia detected by FCM. Mean values with standard deviations of proportions of germinated conidia are presented. Linear Regression curve in marked in red, R^2^ = 9.3.

Conidia from stressed cultures were collected using a cotton swab into a 10 × phosphate-buffered saline solution (PBS, 1.370 M NaCl, 27 mM KCl, 0.1 M Na_2_PO_4_, 18 mM KH_2_PO_4_), pH 7.4, and their concentration was adjusted to 5–7 × 10^6^ conidia/ml. Propidium iodide (PI) was added to attain the final concentration of 2 μg/ml. Samples were incubated with PI for 30 min at room temperature. Three technical replicates per sample were measured using an LSRII (Becton Dickinson, New Jersey, USA) flow cytometer equipped with FACSdiva 6 Software at the Service Centre for Cytometry and Microscopy at the Institute of Microbiology of the Czech Academy of Sciences. Excitation was performed using a Melles Griot 85-YCA-025 laser (23 mW) with a wavelength of 561 nm. Fluorescence was collected through a 590 LP filter and a 610/20 BP filter. Data were analysed in FlowJo 7.6.1 (Tree Star, Inc., Ashland, USA). We verified the precision of this method by testing the viability test of conidia (1) obtained from a non-stressed culture grown under convenient conditions, (2) conidia fixed with 70% ethanol, and (3) a mixture of non-stressed and fixed conidia in various proportions. The mean percentages of viable conidia from untreated and fixed cultures were 90% and 3.8%, respectively. The detected percentages of viable conidia corresponded to the predefined proportions of non-stressed and fixed conidia. We therefore consider flow cytometry with PI staining a suitable method for testing conidial viability. As the viability does not mean germinability, we also cultivated these samples on GYEA for 14 days. Each sample was cultivated at three different conidial concentrations in three replications. Linear Regression Analysis revealed strong correlation between the mean proportions of germinating conidia and proportions of viable conidia detected by FCM (Fig. 5), R^2^ = 9.3. The proportion of germinated conidia was lower than proportion of viable conidia detected by FCM, only 36% of non-stressed conidia are able to germinate. Thus, *P. destructans* conidia have relatively low natural germinability or they need specific cultivation conditions for germination. The germination process is a complex result of environmental and physiological factors^64^. Thus, we cannot foresee the germination efficiency of stressed conidia in nature. However, observed correlation between conidial in vitro germination and conidial viability tests enables insight into approximate germination potential of conidia. Data analysis and gating strategy is presented in Supplementary Figure S1 online.

### Statistics

Biolog data were visualized using PCA (Principal Component Analysis) in PAST^65^. The statistical significance of the type of ecology was evaluated by one-way NPMANOVA with Bonferroni-corrected *p*-values using Bray–Curtis distance and 9,999 permutations. Differences in SA, SR and H between ecological groups were evaluated using Kruskal–Wallis statistics supplemented with Mann–Whitney pairwise comparison and Bonferroni correction. Significance of medium alkalization by urea degradation was computed by permutation test using 9999 permutations.

## Supplementary information

Supplementary information 1.

Supplementary information 2.

## Data Availability

All primary data are presented in Supplementary material.
