# Supplementary material for: Comparative eco-physiology revealed extensive enzymatic curtailment, lipases production and strong conidial resilience of the bat pathogenic fungus Pseudogymnoascus destructans
Source: Sci Rep. 2020 Oct 5;10:16530. doi: 10.1038/s41598-020-73619-7 (PMC7536203; doi:10.1038/s41598-020-73619-7)
Supplement: Supplementary file 1 — Supplementary information 1. [file 41598_2020_73619_MOESM1_ESM.pdf]

Title: Comparative eco-physiology revealed extensive enzymatic curtailment, lipases production and strong conidial resilience of the bat pathogenic fungus *Pseudogymnoascus destructans*

Authors: Tereza Veselská[1,2], Karolína Homutová[1], Paula García Fraile[1], Alena Kubátová[2], Natália Martínková[3], Jiří Pikula[4], Miroslav Kolařík[1*]

Affiliations: [1] Laboratory of Fungal Genetics and Metabolism, Institute of Microbiology, Czech Academy of Sciences (CAS), Vídeňská 1083, CZ-14220 Prague, Czech Republic

[2] Department of Botany, Faculty of Science, Charles University in Prague, Benátská 2, CZ-12801 Prague, Czech Republic

[3] Institute of Vertebrate Biology, Czech Academy of Sciences, Květná 8, CZ-60365 Brno, Czech Republic

[4] Department of Ecology and Diseases of Game, Fish and Bees, University of Veterinary and Pharmaceutical Sciences Brno, Palackého třída 1946/1, CZ-61242 Brno, Czech Republic

[*]**Corresponding author:** Miroslav Kolařík, Fax: +420 241 062 347, phone: Institute of Microbiology (+420) 29644 2332, E-mail: mkolařík@biomed.cas.cz

Table S1: The most utilised nutrient sources on each Biolog MicroPlates tested.

Nutrient sources	*P. destructans*	Cave fungi	Dermatophytes
Carbon	Tween 80	D-Trehalose	L-Phenylalanine
	D-Mannose	Gentibiose	L-Glutamic Acid
	Gentibiose	α-D-Glucose	L-Alanine
	D-Trehalose	D-Fructose	Glycyl-L-Glutamic Acid
	D-Fructose	N-Acetyl-D-Glucosamine	N-Acetyl-D-Glucosamine
	α-D-Glucose	Maltose	L-Analyl-Glycine
	Glycogen	D-Mannose	L-Ornitine
	L-Glutamic Acid	D-Psicose	α-D-Glucose
	Turanose	Sucrose	D-Mannose
	D-Psicose	Glycerol	Alaninamid
Nitrogen	Gly-Gln	Guanosine	Ala-Glu
	Urea	L-Arginine	Gly-Glu
	Guanosine	Urea	L-Citrulline
	Gly-Asn	Gly-Asn	Ala-Gln
	Gly-Glu	Ala-Gly	Gly-Gln
	allantoin	Gly-Gln	Ala-Leu
	Ala-Gly	L-Alanine	Ala-Gly
	L-Glutamine	Uric Acid	L-Ornithine
	Ammonia	Ala-Leu	L-Arginine
	Uric Acid	N-Acetyl-D-Glucosamine	L-Glutamine
Sulphur + Phosphorus	Cytidine-2-Monophosphate	Cytidine-2-Monophosphate	O-Phospho-D-Tyrosine
	O-Phosphoryl-Ethanolamine	O-Phosphoryl-Ethanolamine	O-Phospho-L-Tyrosine
	O-Phospho-L-Serine	Thymidine-3-Monophosphate	Tripolyphosphate
	6-Phospho-Gluonic Acid	Guanosine-2-Monophosphate	Cytidine-2-Monophosphate
	Guanosine-2-Monophosphate	Adenosine-5-Monophosphate	O-Phospho-L-Serine
	2-Deoxy-D-Glucose 6-Phosphate	Guanosine-5-Monophosphate	Phosphoenol Pyruvate
	D-3-Phospho-Glyceric Acid	Cytidine-5-Monophosphate	Guanosine-2-Monophosphate
	Carbamyl Phosphate	Phosphocreatine	Guanosine-5-Monophosphate
	Uridine-5-Monophosphate	D-2-Phospho-Glyceric Acid	Adenosine-2-Monophosphate
	Guanosine-5-Monophosphate	Cytidine-3-Monophosphate	Guanosine-2-,3-Cyclic Monophosphate
Nutrient Supplements	L-Lysine	Orotic Acid	Tween 20
	L-Leucine	Pyridoxal	D,L-Mevalonic Acid
	L-Valine	D-Alanine	β-Nicotinamide Adenine Dinucleotide
	Orotic Acid	D-Glutamic Acid	Folic Acid
	L-Isoleucine + L-Valine	L-Cysteine	D-(+)-Glucose
	D,L-α,δ-Diamino-Pimelic Acid	Pyridoxamine	Menadlone
	D-Alanine	Adenine	(δ) 4-Amino-Imidazole-4(5)-Carboxamide
	L-Tyrosine	Phenylalanine	Inosine + Thiamine
	L-Cysteine	L-Methionine	p-Amino-Benzoic Acid
	Trans-4-Hydroxy L-Proline	L-Valine	Orotic Acid

Table S2: Medium alkalisation by urea degradation.

Fungal strains	Sabouraud medium			Sabouraud medium with urea		
	A	B	C	A	B	C
Pseudogymnoascus destructans 20631-21	6.04	5.87	6.04	8.26	8.29	8.41
P. destructans CCF3941	4.98	4.86	4.86	7.85	7.94	7.38
P. destructans CCF3943	5.78	5.66	6.21	7.53	7.26	7.6
P. destructans CCF4103	5.4	5.32	5.3	8.04	7.86	7.72
P. destructans CCF4986	6.69	6.54	6.39	8.77	8.35	8.75
P. destructans CCF4987	5.52	5.71	5.71	7.43	7.19	7.51
P. sp. 1 CCF5025	5.67	5.56	5.81	7.9	8.54	8.55
P. sp. 1 AK51/11	5.95	6.00	5.96	8.89	8.75	8.74
P. sp. 2 CCF5027	5.05	5.07	5.06	8.84	8.83	8.89
P. sp. 2 CCF5029	4.39	5.4	5.29	8.89	8.92	8.83
P. sp. 2 CCF5030	6.06	5.29	5.41	8.84	8.84	8.84
P. sp. 3 CCF5026	5.7	5.66	5.8	8.75	8.79	8.74
Aspergillus flavus CCF3154	8.52	8.52	7.59	8.39	8.37	8.25
A.luchvensis CCF3984	6.66	6.61	6.54	8.13	7.89	8.1
P. oxalicum 2315T	4.46	4.5	4.5	5.36	5.65	5.67
Arthroderma uncinatum CCF2907	7.74	7.5	7.79	5.46	5.37	5.13
Microsporum canis PL1077/16	7.26	7.37	7.44	9.01	8.14	8.99
M. gypseum CCF4749	7.82	7.54	7.55	9.08	9.09	8.96
Trichophyton interdigitale CCF4473	8.31	8.35	8.38	8.58	7.54	8.5
T. terrestre DMF3040/13	7.61	7.67	7.67	8.75	8.28	9.03
Sterile media	5.58	5.6	5.61	5.78	5.72	5.74

A, B, C – three replication for each strain analysed.

Table S3: Percentage of viable conidia for *Pseudogymnoascus destructans*, other cave *P.* species, and outdoor saprotroph *Aspergillus* species.

stressful condition	day	*Pseudogymnoascus destructans* strains										*Pseudogymnoascus* species					*Aspergillus* species			
		CCF 3938	CCF 3941	CCF 3943	CCF 4103	CCF 4124	CCF 4129	CCF 4131	CCF 4132	Mean	SD	AK 77/11	AK 51/11	AK 71/11	Mean	SD	CCF 3154	CCF 3984	Mean	SD
control	2	90.0	91.0	NA	NA	NA	NA	NA	92.0	91.0	1.0	94.5	78.3	95.0	89.3	9.5	90.1	93.0	91.6	1.5
	7	88.9	77.3	81.1	84.7	86.8	85.3	86.8	86.2	84.6	3.7	94.0	76.4	91.6	87.3	9.5	87.4	91.2	89.3	1.9
	14	78.9	82.6	78.5	82.9	86.3	85.8	84.4	76.2	81.9	3.7	76.1	78.9	93.0	82.7	9.1	70.2	93.6	81.9	11.7
	21	85.4	84.6	78.6	80.2	82.5	70.8	84.2	85.6	81.5	5.0	88.9	69.7	80.0	79.5	9.6	88.7	90.3	89.5	0.8
	28	84.9	74.3	80.9	81.6	88.3	79.4	84.4	80.8	81.8	4.2	72.9	67.8	93.8	78.2	13.7	89.9	93.2	91.6	1.7
	35	83.5	68.0	78.5	80.2	88.4	77.4	79.9	74.4	78.8	6.0	77.2	67.3	95.8	80.1	14.5	89.4	76.5	82.9	6.5
	42	86.6	72.1	79.1	76.6	83.2	83.2	82.1	86.5	81.2	5.0	63.6	57.1	94.8	71.8	20.1	69.9	89.4	79.6	9.7
UVA,B	2	76.1	NA	NA	NA	NA	NA	NA	86.2	81.2	7.1	88.1	53.5	92.7	78.1	21.4	81.5	92.6	87.1	5.5
	7	74.1	NA	85.7	73.3	89.5	NA	82.4	84.4	81.6	6.5	89.6	75.6	86.9	84.0	7.5	86.3	87.6	87.0	0.6
	14	70.9	NA	43.7	53.0	77.3	NA	43.8	72.5	60.2	15.2	86.6	38.2	85.7	70.2	27.7	85.0	85.1	85.0	0.1
	21	69.3	NA	54.5	32.2	57.7	NA	56.6	81.3	58.6	16.4	58.9	34.3	76.2	56.5	21.0	91.2	86.1	88.7	2.6
	28	74.0	NA	59.8	40.8	81.1	NA	69.3	82.0	67.8	15.6	76.9	40.7	84.0	67.2	23.2	75.6	93.7	84.7	9.0
	35	65.7	NA	44.7	25.2	48.3	NA	38.4	57.8	46.7	14.3	75.4	30.5	80.4	62.1	27.5	80.5	94.2	87.4	6.9
	42	76.5	NA	8.1	38.8	NA	NA	46.0	79.6	49.8	29.5	64.2	55.6	88.8	69.5	17.2	75.5	87.1	81.3	5.8
light	2	82.0	76.5	NA	NA	NA	NA	NA	90.2	82.9	6.9	67.0	70.3	96.2	77.8	13.0	84.4	94.5	89.4	5.1
	7	93.5	74.3	52.5	NA	87.0	89.1	87.6	89.1	81.9	14.2	82.2	76.8	92.5	83.9	6.5	84.5	88.3	86.4	1.9
	14	91.9	76.2	66.3	NA	87.5	86.2	83.7	67.9	80.0	10.0	88.4	75.4	92.7	85.5	7.3	76.1	72.8	74.5	1.7
	21	86.3	54.7	76.6	NA	75.3	76.7	77.0	88.2	76.4	10.9	84.1	79.9	89.2	84.4	3.8	90.8	87.3	89.1	1.7
	28	83.3	48.2	76.0	NA	73.1	84.2	81.0	84.8	75.8	12.9	74.0	69.4	94.4	79.3	10.9	87.3	94.2	90.8	3.5
	35	87.4	36.1	71.2	NA	28.6	82.6	72.8	61.8	62.9	22.5	86.3	50.0	95.0	77.1	19.5	70.7	91.6	81.2	10.4
	42	86.4	27.3	71.4	NA	63.1	61.9	64.3	76.5	64.4	18.6	87.6	80.6	77.3	81.8	4.3	78.7	91.8	85.3	6.6
UVA	2	82.5	65.3	NA	NA	NA	NA	NA	87.7	78.5	11.7	93.5	72.7	94.7	87.0	10.1	84.2	95.5	89.8	5.7
	7	93.2	67.9	71.0	92.7	92.4	90.9	86.5	84.3	84.9	10.1	86.5	71.0	89.5	82.3	8.1	87.3	92.5	89.9	2.6
	14	88.6	32.5	52.2	41.9	78.0	81.8	80.3	65.4	65.1	20.7	91.1	82.6	91.8	88.5	4.2	74.7	86.6	80.6	6.0
	21	79.5	22.8	54.8	10.2	67.3	71.2	48.8	74.6	53.6	25.2	75.8	71.5	83.6	77.0	5.0	86.4	82.9	84.6	1.8
	28	70.7	19.4	60.3	72.2	50.1	83.9	48.6	78.8	60.5	20.9	84.6	42.7	92.6	73.3	21.9	89.8	92.8	91.3	1.5
	35	69.8	2.6	58.8	50.9		58.3	44.4	58.6	49.0	21.9	59.3	23.8	90.5	57.9	27.2	84.9	85.5	85.2	0.3
	42	60.8	14.2	57.9	73.5	70.1	43.5	77.4	71.5	58.6	21.0	81.9	10.8	76.1	56.3	32.2	75.4	91.4	83.4	8.0
30 °C	2	73.3	73.7	NA	NA	NA	NA	NA	86.3	77.8	7.4	91.5	78.7	95.9	88.7	7.3	88.8	94.1	91.5	2.7
	7	89.0	65.9	81.1	69.3	82.4	77.1	68.6	83.2	77.1	8.3	89.3	67.9	91.9	83.0	10.8	81.1	77.1	79.1	2.0
	14	92.1	58.9	61.2	67.4	80.2	79.5	76.7	57.8	71.7	12.3	77.0	74.5	81.9	77.8	3.1	79.3	78.7	79.0	0.3
	21	91.1	50.6	67.0	87.3	47.0	79.5	72.2	69.9	70.6	15.8	53.2	70.5	77.8	67.2	10.3	88.1	87.8	88.0	0.1
	28	80.4	34.6	72.2	85.0	77.0	83.5	64.9	79.6	72.2	16.5	57.1	3.3	79.5	46.7	32.0	88.6	95.4	92.0	3.4
	35	82.3	42.1	76.6	72.4	77.9	78.3	60.4	73.2	70.4	13.1	48.0	2.5	84.4	45.0	33.5	84.3	85.0	84.6	0.4
	42	86.1	18.1	39.2	46.9	68.1	53.1	53.1	67.1	53.9	20.6	47.2	7.0	78.1	44.1	29.1	79.2	92.4	85.8	6.6
37°C	2	81.1	81.1	NA	NA	NA	NA	NA	88.2	83.5	4.1	78.4	70.7	95.6	81.6	10.4	95.2	95.4	95.3	0.1

	7	78.1	78.1	80.2	NA	37.4	73.9	NA	83.2	71.8	17.1	41.8	76.8	92.7	70.4	21.3	82.8	93.3	88.1	5.3
	14	37.5	37.5	52.9	NA	31.9	31.2	NA	26.0	36.2	9.3	61.1	12.0	23.7	32.2	20.9	81.4	83.1	82.3	0.8
	21	1.6	1.6	7.1	NA	3.9	3.2	NA	2.9	3.4	2.1	0.7	3.3	2.9	2.3	1.1	81.2	88.6	84.9	3.7
	28	1.0	1.0	9.0	NA	9.5	5.5	NA	6.2	5.4	3.7	2.1	3.3	4.3	3.2	0.9	90.4	94.7	92.5	2.2
	35	1.2	1.2	3.5	NA	6.3	4.8	NA	2.8	3.3	2.0	7.8	2.1	2.7	4.2	2.5	77.7	94.0	85.8	8.1
	42	1.0	1.0	2.0	NA	9.5	3.0	NA	6.4	3.8	3.4	7.5	3.9	7.5	6.3	1.7	75.8	91.3	83.5	7.7
dryness	2	71.1	63.1	NA	NA	NA	NA	NA	87.6	73.9	12.5	46.4	52.8	92.4	63.9	20.4	69.6	93.1	81.4	11.8
	7	84.2	21.3	63.5	80.6	71.3	79.5	60.2	74.3	66.9	20.2	77.0	59.7	89.5	75.4	12.2	80.3	93.8	87.0	6.7
	14	86.0	18.4	53.5	80.6	71.4	66.9	65.1	71.4	64.2	20.9	71.5	64.6	85.1	73.8	8.5	66.1	83.1	74.6	8.5
	21	69.3	6.8	51.4	87.8	28.8	79.1	45.3	77.3	55.7	28.0	63.5	53.7	85.7	67.7	13.4	79.3	84.8	82.1	2.8
	28	80.3	7.3	46.6	76.8	62.1	72.2	65.2	73.7	60.5	24.0	78.4	49.8	93.0	73.7	18.0	91.1	91.2	91.2	0.0
	35	76.1	15.7	50.1	71.8	40.3	73.8	60.6	84.2	59.1	22.7	68.7	64.1	89.6	74.1	11.1	81.2	92.8	87.0	5.8
	42	72.0	7.4	46.9	76.8	74.2	67.7	61.7	78.3	60.6	23.8	71.1	70.8	87.0	76.3	7.6	62.5	88.7	75.6	13.1
25°C	2	84.5	73.6	NA	NA	NA	NA	NA	91.3	83.1	8.9	94.0	72.9	95.0	87.3	10.2	84.5	90.5	87.5	3.0
	7	87.3	64.0	NA	77.1	92.7	92.6	71.8	85.9	81.6	11.0	85.6	73.1	87.2	82.0	6.3	87.8	93.3	90.5	2.8
	14	89.0	62.0	NA	83.2	76.6	87.7	52.1	75.2	75.1	13.7	88.2	77.5	92.9	86.2	6.5	79.4	84.6	82.0	2.6
	21	89.0	79.4	NA	82.0	90.9	84.4	71.0	85.8	83.2	6.7	82.9	77.0	91.9	83.9	6.1	92.7	86.1	89.4	3.3
	28	66.1	73.1	NA	69.1	91.2	89.2	76.2	84.9	78.5	9.9	79.3	71.8	95.0	82.0	9.7	94.8	93.0	93.9	0.9
	35	80.9	48.9	NA	68.1	87.3	83.2	64.3	67.9	71.5	13.3	80.6	59.6	94.0	78.1	14.2	74.6	92.9	83.8	9.1
	42	90.7	58.5	NA	76.8	87.7	84.8	71.4	83.2	79.0	11.2	83.3	72.1	92.9	82.8	8.5	74.6	89.7	82.1	7.5
34 °C	2	NA	NA	NA	NA	NA	NA	NA	NA	NA	NA	NA	NA	NA	NA	NA	NA	NA	NA	NA
	7	88.9	58.0	70.5	83.1	82.8	80.4	51.9	77.6	74.1	13.0	NA	NA	NA	NA	NA	NA	NA	NA	NA
	14	51.7	20.1	86.0	81.7	83.0	85.8	82.0	35.0	65.7	26.3	NA	NA	NA	NA	NA	NA	NA	NA	NA
	21	36.9	9.0	62.9	59.2	52.3	58.7	69.9	10.5	44.9	23.7	NA	NA	NA	NA	NA	NA	NA	NA	NA
	28	30.9	3.8	27.6	38.6	24.7	11.5	19.7	6.0	20.3	12.4	NA	NA	NA	NA	NA	NA	NA	NA	NA
	35	4.1	8.5	9.5	14.4	16.6	4.3	18.4	2.9	9.8	6.0	NA	NA	NA	NA	NA	NA	NA	NA	NA
	42	1.8	6.1	4.6	1.7	3.4	2.2	1.7	5.5	3.4	1.8	NA	NA	NA	NA	NA	NA	NA	NA	NA
34/20 °C	2	NA	NA	NA	NA	NA	NA	NA	NA	NA	NA	NA	NA	NA	NA	NA	NA	NA	NA	NA
	7	88.2	NA	62.5	88.7	78.4	81.9	57.8	89.6	78.1	13.0	NA	NA	NA	NA	NA	NA	NA	NA	NA
	14	86.4	NA	46.2	91.9	83.5	89.0	83.3	78.6	79.8	15.4	NA	NA	NA	NA	NA	NA	NA	NA	NA
	21	76.6	NA	86.4	73.7	63.0	83.9	70.2	46.2	71.4	13.7	NA	NA	NA	NA	NA	NA	NA	NA	NA
	28	67.2	NA	83.0	88.5	82.1	77.8	68.9	53.3	74.4	12.1	NA	NA	NA	NA	NA	NA	NA	NA	NA
	35	81.1	NA	75.5	78.0	75.2	73.5	55.5	45.7	69.2	13.3	NA	NA	NA	NA	NA	NA	NA	NA	NA
	42	83.3	NA	77.4	87.2	61.7	82.2	63.5	26.7	68.8	21.0	NA	NA	NA	NA	NA	NA	NA	NA	NA

NA – not available

Figure S1: Data analyses from flow cytometry – gating strategy.

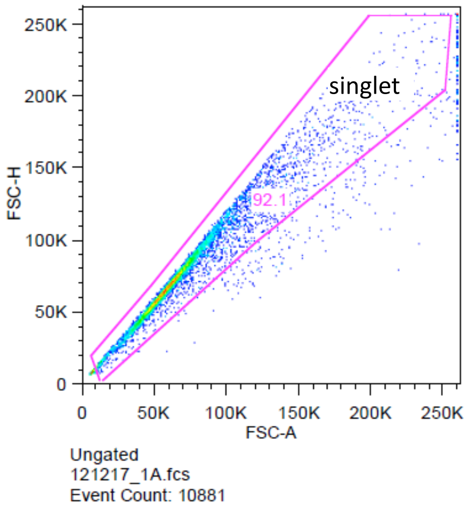

Data was first checked on FSC-A – FSC-H plot to gate population “singlet” which ensures that only separated conidia will be analyzed and all conidial clusters will be omitted.

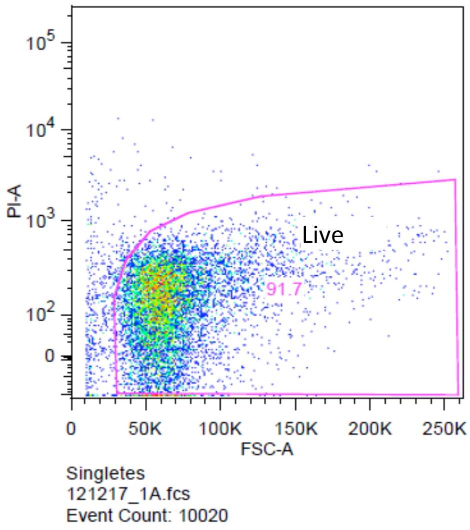

All events in “singlet” population with PI fluorescence under threshold (defined by measurements of unlabeled conidia) were gated to population “Live” which contains living conidia with negative PI fluorescence.

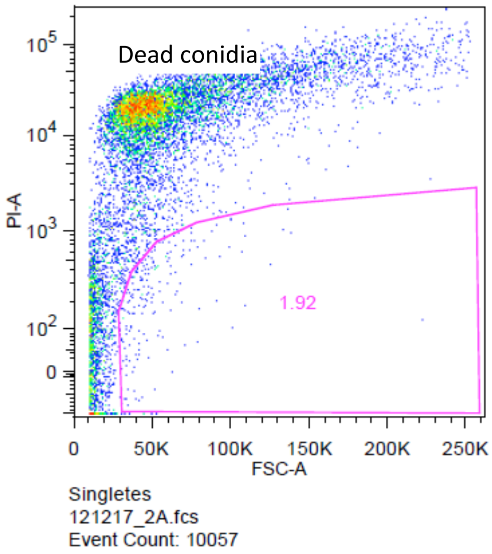

All events with higher fluorescence intensities than defined threshold represent dead conidia, as PI can stains only dead cells.
